# Lateral Hypothalamic Area Glutamatergic Neurons and Their Projections to the Lateral Habenula Modulate the Anesthetic Potency of Isoflurane in Mice

**DOI:** 10.1007/s12264-021-00674-z

**Published:** 2021-04-13

**Authors:** Shiyi Zhao, Rui Li, Huiming Li, Sa Wang, Xinxin Zhang, Dan Wang, Juan Guo, Huihui Li, Ao Li, Tingting Tong, Haixing Zhong, Qianzi Yang, Hailong Dong

**Affiliations:** Department of Anesthesiology and Perioperative Medicine, Xijing Hospital, Fourth Military Medical University, Xi’an, 710032 China

**Keywords:** Anesthesia, Isoflurane, Lateral hypothalamic area, Lateral habenula, Glutamatergic neuron

## Abstract

The lateral hypothalamic area (LHA) plays a pivotal role in regulating consciousness transition, in which orexinergic neurons, GABAergic neurons, and melanin-concentrating hormone neurons are involved. Glutamatergic neurons have a large population in the LHA, but their anesthesia-related effect has not been explored. Here, we found that genetic ablation of LHA glutamatergic neurons shortened the induction time and prolonged the recovery time of isoflurane anesthesia in mice. In contrast, chemogenetic activation of LHA glutamatergic neurons increased the time to anesthesia and decreased the time to recovery. Optogenetic activation of LHA glutamatergic neurons during the maintenance of anesthesia reduced the burst suppression pattern of the electroencephalogram (EEG) and shifted EEG features to an arousal pattern. Photostimulation of LHA glutamatergic projections to the lateral habenula (LHb) also facilitated the emergence from anesthesia and the transition of anesthesia depth to a lighter level. Collectively, LHA glutamatergic neurons and their projections to the LHb regulate anesthetic potency and EEG features.

## Introduction

The lateral hypothalamic area (LHA) has been widely reported to regulate consciousness transition. There are four types of neurons in the LHA: orexinergic, GABAergic, melanin-concentrating hormone (MCH), and glutamatergic neurons. The orexinergic neurons, exclusively located in the LHA, are known for their pro-arousal effect in both sleep and anesthesia, with little influence on the transition from wakefulness to unconsciousness after administration of general anesthesia [1–5]. The GABAergic neurons in the LHA are active when awake and during rapid eye movement (REM) sleep. Activation of LHA GABAergic neurons promotes wakefulness by inhibiting sleep-promoting neurons within the ventrolateral preoptic nucleus and thalamic reticular nucleus [6–8]. The MCH neurons are active in REM sleep and sleep-promoting [9, 10]. Activation of MCH neurons is sufficient to trigger the transition from non-REM (NREM) to REM sleep and increase the duration of REM sleep [11, 12]. However, the involvement of LHA glutamatergic neurons in the regulation of consciousness, especially during anesthetic procedures, has not yet been fully elucidated.

The glutamate level in the LHA has been reported to increase quickly in the active waking and REM sleep periods but not the NREM sleep state [13]. Microinjection of glutamate into the LHA area lengthens the time rats spend in waking and concomitantly decreases the time in REM and NREM sleep [14]. LHA glutamatergic neurons send dense projections to the lateral habenula (LHb) [15]. This pathway has been reported to regulate multiple physiological functions, such as feeding, reward, and taste sensitivity [15, 16]. LHb neurons are involved in propofol anesthesia: blocking the output of LHb glutamatergic neurons prevents the propofol-induced loss of muscle tone and enhancement of EEG power [17].

In the present study, we combined pharmacogenetic ablation techniques and optogenetics with “designer receptors exclusively activated by designer drugs” (DREADDs) to explore the function of LHA glutamatergic neurons and their projections to the LHb in isoflurane anesthesia.

## Materials and methods

### Animals

Vglut2-Cre mice were purchased from the Jackson Laboratory. All animals were housed under specific-pathogen-free conditions with a constant temperature of 22°C – 24°C and humidity of 38% – 42% on a light-controlled schedule (lights on 07:00– 19:00) with *ad libitum* access to food and water. All experiments were done during the light-on period. The experimental protocol was approved by the Ethics Committee for Animal Experimentation and conducted in accordance with the Guidelines for Animal Experimentation at the Fourth Military Medical University as well as the ARRIVE guidelines. Sample size was calculated based on the statistical formula and previous reports [18].

### Surgical procedure

Mice were fixed in a stereotaxic frame (RWD, Shenzhen, China) under 1.4%–1.5% isoflurane anesthesia with oxygen (flow rate 1.0 L/min), and the eyes were protected by erythromycin ophthalmic ointment. After shaving and skin antisepsis, the scalp was locally anesthetized with 1% lidocaine followed by a sagittal incision. During the surgery, mice were constantly kept warm with a heating mat. Viruses (Brain-VTA, Wuhan, China) were then microinjected into the LHA. For pharmacogenetic ablation tests, 200 nL rAAV-Ef1α-DIO-taCasp3-TEVP (Caspase 3) or rAAV- Ef1α-DIO-mCherry (Control) were bilaterally microinjected into the LHA (AP –1.80 mm, ML ±0.9 mm, DV –5.1 mm) at 50 nL/min. For chemogenetic tests, 200 nL AAV-Ef1α-DIO-hM3Dq-mCherry (hM3Dq), AAV-Ef1α-DIO-hM4Di-mCherry (hM4Di), or rAAV-Ef1α-DIO-mCherry (Control) were bilaterally microinjected into the LHA. After injection, the micropipette was left in place for 10 min followed by slow retrieval. For optogenetic activation tests, 200 nL rAAV-Ef1α-DIO-hChR2-mCherry (ChR2) or control virus was unilaterally injected into the LHA (AP –1.80 mm, ML +0.9 mm, DV –5.1 mm) for the activation experiments, followed by the placement of an optical fiber (Inper Ltd, Hangzhou, China) into the unilateral LHA (AP –1.80 mm, ML +0.9 mm, DV –5.0 mm) or ipsilateral LHb (AP –1.70 mm, ML +0.5 mm, DV –2.75 mm). For the optogenetic inhibition tests, rAAV-Ef1α-DIO-NpHR-mCherry (NpHR) or control virus was bilaterally injected into the LHA, followed by the placement of optical fibers into the LHA on both sides (AP –1.80 mm, ML ±0.9 mm, DV –5.0 mm) or the LHb on both sides (AP –1.70 mm, ML ± 0.95 mm, DV –2.95 mm, at a 10° angle towards the midline). After virus injection, three stainless-steel screws were anchored to the skull as electroencephalogram (EEG) electrodes: the positive electrode on one side of the head (AP –1.5 mm, ML +1.5 mm), the negative electrode on the other side (AP +1.5 mm, ML –1.5 mm), and a reference electrode at the back of the head (AP –5.5 mm, ML 0 mm). We used unipolar EEG electrodes for recording. The mouse brain atlas by Paxinos and Franklin was used to determine the stereotaxic coordinates and bregma was used as the stereotactic reference point during surgery [19]. Optical fibers and skull screws were fixed with methyl methacrylate cement. The mice were then moved to a warming pad until they recovered consciousness. Meloxicam (0.03 mg/kg) was used for post-operative analgesia for 3 days. Mice were allowed to recover for 3 weeks before the behavioral assessments.

After experiments, we excluded the mice with failure of optical fiber implantation or virus expression. In the pharmacogenetic ablation and chemogenetic experiments, no animal was removed. In the experiments using optical stimulation of LHA glutamatergic cell bodies, we removed one animal from the ChR2-mCherry group for incorrect placement of the optical fiber. We also removed one mouse from the ChR2-mCherry group for poor virus expression in the experiments using optical stimulation of LHA glutamatergic terminals.

### Immunohistochemistry

Deeply anesthetized with isoflurane, mice were transcardially perfused with saline followed by ice-cold 4% paraformaldehyde. Brains were post-fixed in paraformaldehyde for 2 h at room temperature and then immersed in 30% sucrose overnight for dehydration. The brains were coronally cut into 40-μm sections on a freezing microtome (Leica CM1900, Germany). Sections containing the LHA were rinsed in phosphate-buffered saline (PBS, pH 7.4) three times for 10 min each and blocked by 5.0% normal donkey serum (NDS) with 0.3% triton-X100 in PBS (PBST) for 2 h at room temperature. Then the sections were incubated with anti-glutamate antibody (1:500, G6642, Sigma-Aldrich, USA) in 2.5% NDS with PBST for 24 h at 4°C. Afterwards, the sections were washed three times with PBS, and incubated with Alexa Fluor 488 (1:500 diluted in 2.5% NDS with PBST; 715–545–150, Jackson ImmunoResearch, USA) for 2 h at room temperature. Finally, the sections were washed, mounted, cover-slipped, and imaged using a laser confocal fluorescence microscope (VS120, Olympus, Japan).

### Examination of Induction and Emergence Times

After 30 min of habituation in a horizontal cylinder (12 cm in diameter), mice were anesthetized with 1.4% isoflurane in 100% O_2_ at 1.0 L/min. The cylinder was rotated 90° every 10 s until the mouse could not turn prone onto all four limbs, which referred to loss of the righting reflex (LORR). The induction time was defined as the time from the onset of isoflurane inhalation to LORR. The mice were continuously anesthetized for 30 min, and the emergence time was defined as the interval from the cessation of anesthesia to recovery of the righting reflex (RORR) when the supine mouse returned to the prone position. During the experiments, a heating pad was used to control body temperature to approximately 37°C.

For chemogenetic experiments, clozapine N-oxide (CNO, 1 mg/kg; Cayman, China) or an equivalent volume of saline was injected intraperitoneally 30 min before anesthesia. For optogenetic experiments, one train of blue laser pulses (473 nm, 20 Hz, 30 ms, 10 s-ON and 10 s-OFF) or a yellow laser (594 nm, 1 Hz, 1 s, 10 s-ON and 10 s-OFF) was delivered to the animals during induction and emergence. During induction, the opto-stimulation was administered as isoflurane inhalation started, and continued until mice achieved LORR. During emergence, mice were optically stimulated from the cessation of isoflurane inhalation until RORR.

### EEG Recording and Analysis

The EEG signal was continuously recorded using the PowerLab 16/35 amplifier system (PL3516, AD Instruments, New Zealand) and LabChart Pro V8.1.13 software (MLU60/8, AD Instruments). The raw EEG data were collected at 1000 Hz and bandpass filtered at 0.3–50 Hz for further analysis.

To calculate the burst-suppression ratio (BSR, a marker of anesthetic depth [20]), the mice were exposed to 1.0% isoflurane for approximately 30 min when the burst-suppression wave regularly occurred and the BSR became stable at approximately 60%. One-min blue laser (473 nm, 20 Hz, 30 ms, 10–15 mW from tips; Thinker Tech, Nanjing, China) was delivered for activation, while a yellow laser (594 nm, 1 Hz, 1 s, 10 mW from tips; Thinker Tech) was used for inhibition. The EEG voltage threshold was set according to the amplitude of the suppression in the experimental mice themselves. If the amplitude of the EEG was below threshold for >0.5 s, it was defined as a suppression event and assigned a value of 1. Otherwise, signals above the threshold were defined as a burst event and assigned a value of 0. Finally, the BSR was calculated as the percentage of suppression events for 2 min before and during optical stimulation.

For EEG spectral analysis, 0.8% isoflurane anesthesia was delivered for 30 min, and the optical stimulation was given as described above. The spectrogram function provided by the MatLab signal processing toolbox was used to calculate the absolute power spectrum in each time-window. The parameters were set as follows: non-equispaced fast Fourier transform 2048; sampling frequency (Fs) = 1000; windows (window function) = Hanning; no overlap (window overlaps the number of points) = length (windows)/2. The EEG signal was classified into 5 frequency bands by LabChart as follows: delta (δ: 0.3–4 Hz), theta (θ: 4–10 Hz), alpha (α: 10–15 Hz), beta (β: 15–25 Hz), and gamma (γ: 25–50 Hz). The relative power of each frequency band was calculated as the percentage of the total power of 0.3–50 Hz. The total power percentages of frequency bands were calculated to investigate the changes in depth of anesthesia.

### *In vitro* Electrophysiological Recording

Three weeks after viral expression, the brains of Vglut2-Cre mice were collected and immersed in oxygenated (95% O_2_/5% CO_2_) ice-cold artificial cerebrospinal fluid (ACSF) containing (in mmol/L) 124 NaCl, 25 NaHCO_3_, 2.5 KCl, 1 NaH_2_PO_4_, 2 CaCl_2_, 2 MgSO_4_, and 37 glucose. Coronal slices containing the LHA or LHb were cut (300 μm) on a vibratome (Leica VT1200S, Germany) and incubated for 45 min at 37°C in ACSF containing (in mmol/L) 124 NaCl, 24 NaHCO_3_, 3.8 KCl, 1.2 NaH_2_PO_4_, 1 MgCl_2_, 2.5 CaCl_2_, and 10 glucose, saturated with 95% O_2_/5% CO_2_ at pH 7.4. Then, the slices were transferred to the recording chamber and perfused continuously with oxygenated ACSF (1.5–2 mL/min) at room temperature.

Whole-cell recording was performed using micropipettes prepared from borosilicate glass capillaries (1.5 mm OD, 1.1 mm ID) using a horizontal puller (P-97, Sutter Instruments), with resistances of 4–6 MΩ. The pipette solution consisted of (in mmol/L) 130 K-gluconate, 4 KCl, 1 MgCl_2_, 10 hydroxyethyl piperazineethanesulfonic acid, 0.3 egtazic acid, 4 Mg-ATP (adenosine triphosphate), and 0.3 Na-GTP (guanosine-5-triphosphate) (pH 7.4). A current clamp was used to assess the electrophysiological characteristics in response to the activation or inhibition of glutamatergic neurons in the LHA.

### Statistical Analysis

Prism 8.0 (GraphPad Software, USA) was used for statistical analysis. Data are presented as the mean ± standard deviation. The results of righting reflex assessment were analyzed by the unpaired Student’s *t* test in the pharmacogenetic ablation and optogenetics experiments, and two-way ANOVA followed by Bonferroni correction in the chemogenetic test. Differences in the BSR and EEG spectral power before and after optical stimulation were analyzed using paired *t* tests. *P* values <0.05 were considered to be statistically significant in all cases.

## Results

### Selective ablation of LHA glutamatergic neurons facilitates the effect of isoflurane anesthesia

**Fig. 1:**
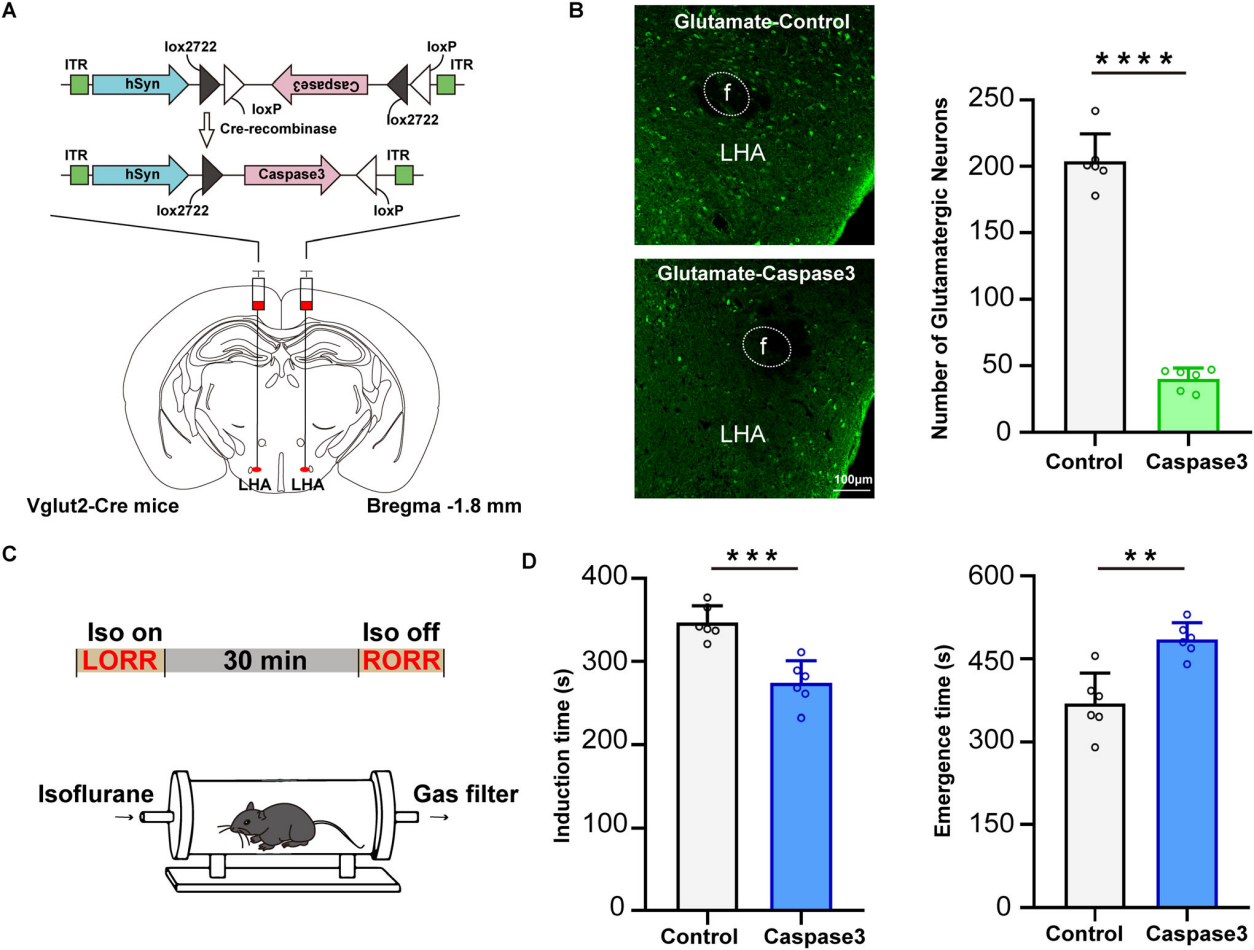
Pharmacogenetic ablation of LHA glutamatergic neurons accelerates the induction and prolongs the emergence of anesthesia. **A** Schematic of virus injection. **B** Left, representative immunofluorescent images (×20) of glutamatergic neurons stained in the Control group (above) and the Caspase 3 group (below); right, numbers of glutamatergic neurons in the Caspase3 and Control groups. **C** Protocol of the righting reflex test. **D** Induction and emergence times in the two groups. Data are shown as the mean ± standard deviation, *n* = 6 per group; **P <0.01, ***P <0.001, ****P <0.0001; f, fornix; Iso, isoflurane; LORR, loss of righting reflex; LHA, lateral hypothalamic area; RORR, recovery of righting reflex.

To explore the role of LHA glutamatergic neurons in the regulation of the anesthesia-arousal transition, a cre-dependent AAV expressing caspase3 was bilaterally injected into the LHA of Vglut2-Cre mice to selectively ablate the glutamatergic neurons (Fig. 1A). The immunochemical results confirmed that this strategy reduced the number of LHA glutamatergic neurons from 203.8 ± 21.0 to 40.0 ± 8.3 (*t*_(10)_ = 17.81, *P* <0.0001, *n* = 6 per group, Fig. 1B). The righting reflex was used to analyze the anesthetic potency (Fig. 1C). Knockdown of LHA glutamatergic neurons markedly shortened the induction time of anesthesia (346.8 ± 20.4 s *vs* 273.8 ± 26.9 s, *t*_(10)_ = 5.289, *P* = 0.0004, *n* = 6 per group, Fig. 1D left), and prolonged the emergence time as well (368.7 ± 55.4 s *vs* 484.8 ± 30.4 s, *t*_(10)_ = 4.501, *P* = 0.0011, *n* = 6 per group, Fig. 1D right).

### Chemogenetic manipulation of LHA glutamatergic neurons affects anesthesia induction and recovery

**Fig. 2:**
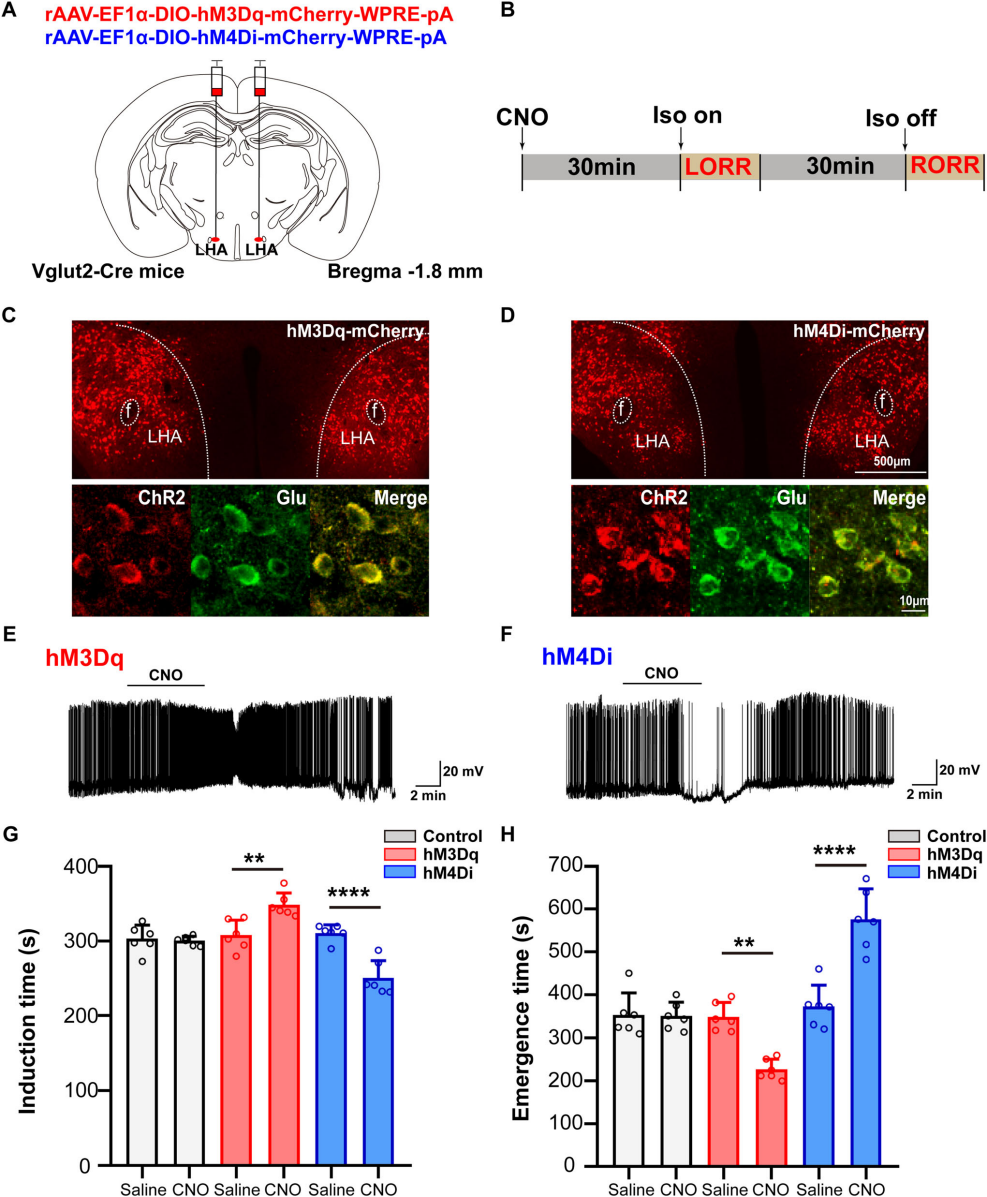
Chemogenetic manipulation of LHA glutamatergic neurons regulates the induction and emergence of isoflurane anesthesia. **A** Schematic of chemogenetic virus injection. **B** Schematic showing the protocol of chemogenetic activation of LHA glutamatergic neurons during isoflurane anesthesia. **C**, **D** Virus expression of hM3Dq (**C**) and hM4Di (**D**) in the LHA. **E**, **F** *Ex vivo* electrophysiological recordings from LHA glutamatergic neurons transfected with hM3Dq virus (**E**) or hM4Di virus (**F**). **G**, **H** Induction time (**G**) and emergence time (**H**) of mice with chemogenetic activation or inhibition of the LHA glutamatergic neurons. Data are shown as the mean ± standard deviation, *n* = 6 per group; **P <0.01, ****P <0.0001; CNO, clozapine N-oxide; f, fornix; Iso, isoflurane; LORR, loss of righting reflex; LHA, lateral hypothalamic area; RORR, recovery of righting reflex.

To modulate the activity of LHA glutamatergic neurons, chemogenetic viruses containing hM3Dq or hM4Di were microinjected into the LHA of Vglut2-Cre mice (Fig. 2A), and CNO was injected intraperitoneally 30 min before anesthesia to activate LHA glutamatergic neurons (Fig. 2B). The accuracy and specificity of virus expression was then confirmed by immunofluorescence (Fig. 2C, D). The *ex vivo* electrophysiology showed that the firing rate of LHA glutamatergic neurons increased after the injection of CNO in the hM3Dq-expressing animals (Fig. 2E), and decreased in the hM4Di group (Fig. 2F).

Chemogenetic activation of LHA glutamatergic neurons significantly lengthened the time from wakefulness to LORR (308.3 ± 20.2 s *vs* 348.8 ± 16.1 s, *t*_(30_ = 4.166, *P* = 0.0036, *n* = 6 per group, Fig. 2G), and accelerated the recovery from anesthesia (348.8 ± 33.3 s *vs* 226.7 ± 23.9 s, *t*_(30)_ = 4.585, *P* = 0.0011, *n* = 6 per group, Fig. 2H). On the contrary, the inhibition of LHA glutamatergic neurons by hM4Di shortened the induction time from 311.2 ± 11.1 s to 251.2 ± 23.0 s (*t*_(30)_ = 6.172, *P* <0.0001, *n* = 6 per group, Fig. 2G), and prolonged the emergence time from 372.7 ± 49.1 s to 575.8 ± 71.2 s (*t*_(30)_ = 7.624, *P* <0.0001, *n* = 6 per group, Fig. 2H).

### Optical activation of LHA glutamatergic neurons reduces the depth of isoflurane anesthesia

**Fig. 3:**
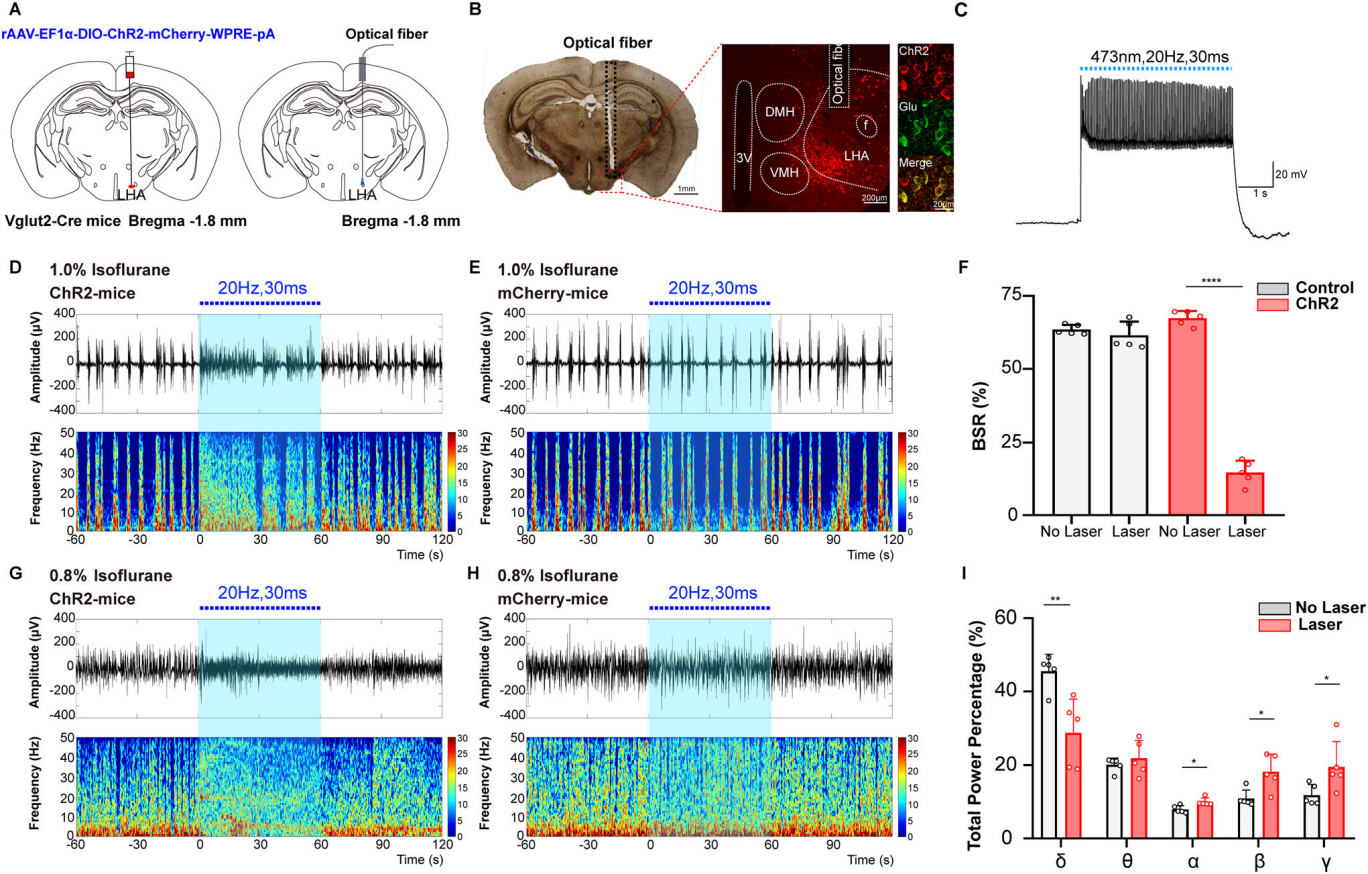
Optogenetic activation of LHA glutamatergic neurons reduces the depth of isoflurane anesthesia. **A** Schematic of excitatory optogenetic virus (ChR2) injection into the LHA in Vglut2-Cre mice. **B** Left, histological images of fiber location in the unilateral LHA; right, immunofluorescent staining showing the specificity of virus expression. **C** *Ex vivo* electrophysiology of ChR2 virus action in LHA glutamatergic neurons. **D**, **E** Representative EEG traces (above) and corresponding power spectra (below) before, during, and after 1 min optical activation under 1.0% isoflurane anesthesia in the ChR2 group (**D**) and the Control group (**E**). **F** Statistics of the change of BSR before and during optical stimulation. **G**, **H** Changes of EEG traces (above) and corresponding power spectra (below) in the ChR2 group (**G**) and the Control group (**H**) under 0.8% isoflurane anesthesia. **I** Comparison of the spectral power percentage for 1 min before and after optical activation in the two groups. Data are shown as the mean ± standard deviation, *n* = 5 per group; *P <0.05, **P <0.01, ****P <0.0001 *vs* control group; BSR, burst-suppression ratio; f, fornix; LHA, lateral hypothalamic area; DMH, dorsomedial hypothalamic nucleus; VMH, ventromedial hypothalamic nucleus; 3V, third ventricle.

**Fig. 4:**
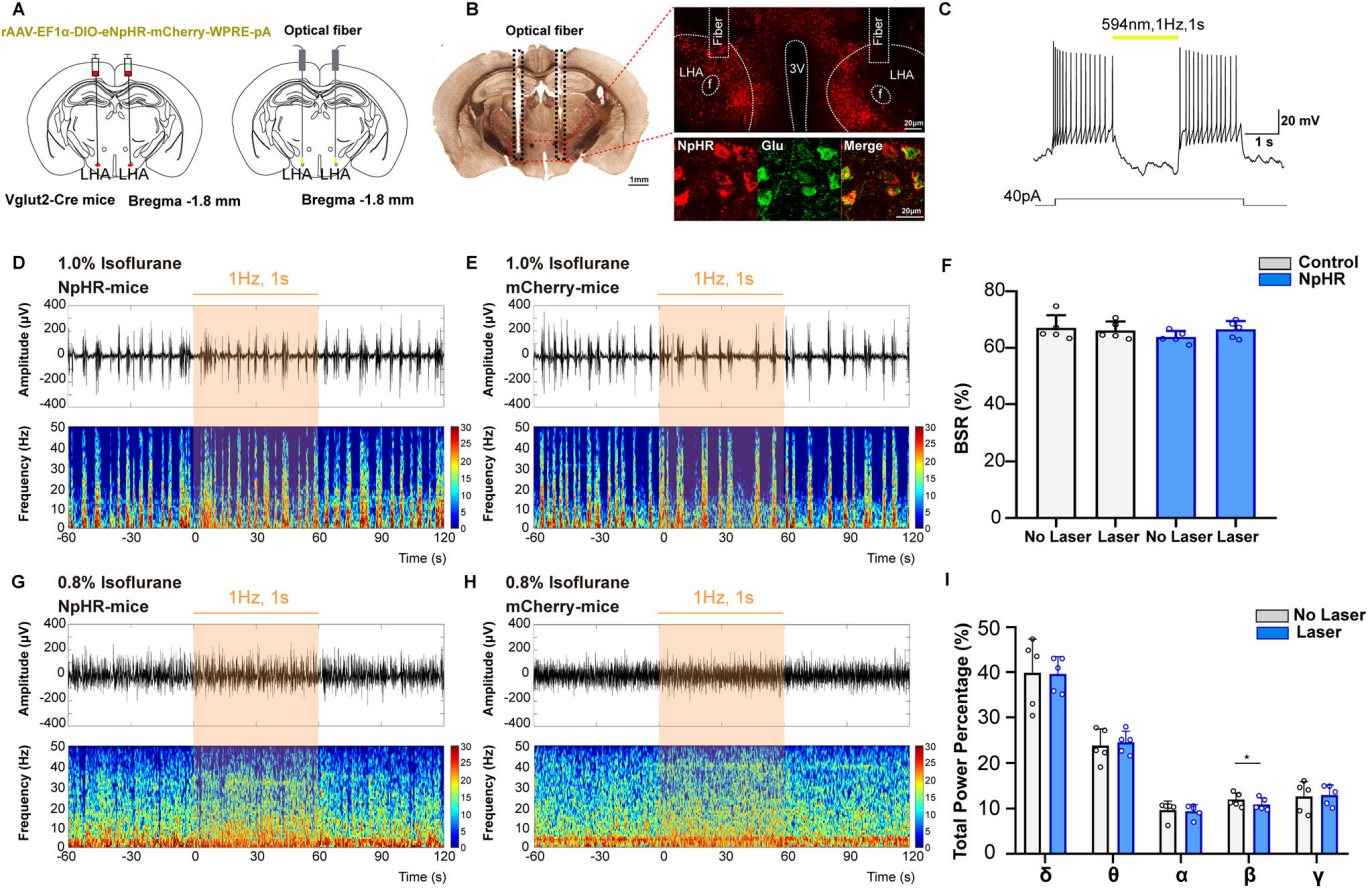
Optical inhibition of LHA glutamatergic neurons slightly changes the power distribution of frequency bands. **A** Schematic of inhibitory optogenetic virus (NpHR) injection into the LHA in Vglut2-Cre mice. **B** Left, histological image showing the location of fibers in the bilateral LHb; right, representative confocal images of immunofluorescent staining. **C** *Ex vivo* electrophysiology of NpHR virus action in LHA glutamatergic neurons. **D**, **E** Representative EEG traces (above) and corresponding power spectra (below) before, during, and after optical inhibition in the NpHR group (**D**) and the Control group (**E**) under 1.0% isoflurane anesthesia. **F** Statistics showing the change of BSR before and during optical stimulation. **G**, **H** Changes of EEG traces (above) and corresponding power spectra (below) in the NpHR group (**G**) and the Control group (**H**) under 0.8% isoflurane anesthesia. I Comparison of the spectral power percentage for 1 min before and after optical activation in the two groups. Data are shown as the mean ± standard deviation, *n* = 5 per group; *P <0.05; BSR, burst-suppression ratio; f, fornix; LHA, lateral hypothalamic area; 3V, third ventricle.

To investigate the regulatory effect of LHA glutamatergic neurons on the depth of anesthesia, burst suppression patterns and power percentages of the frequency bands during anesthesia maintenance were recorded when the optogenetics technique was used to transiently modulate the LHA glutamatergic neuronal spiking [21–23]. The excitatory ChR2-containing virus or inhibitory NpHR-containing virus was injected into the LHA of Vglut2-Cre mice followed by optic fiber implantation (Figs 3A, 4A). The specificity of virus transfection and accuracy of optical fiber location in each animal (Figs 3B, 4B) were confirmed after behavioral experiments. The effectiveness of optical stimulation was successfully tested in *ex vivo* brain slices (Figs 3C, 4C).

Optical activation of LHA glutamatergic neurons significantly changed the EEG pattern under both deep (1.0%, Fig. 3D) and light (0.8%, Fig. 3G) anesthesia in the ChR2 group and not in the control group (Fig. 3E, H). In particular, during the stable maintenance of 1.0% anesthesia, a burst suppression pattern was regularly displayed in the EEG spectrogram, optical stimulation for 1 min resulted in a marked decrease of BSR from 67.4% ± 2.5% to 14.6% ± 4.1% (*t*_(4)_ = 36.58, *P* <0.0001, *n* = 5 per group, Fig. 3F). During the light anesthesia induced by 0.8% isoflurane, no significant burst suppression pattern was observed. Optical activation of LHA glutamatergic neurons reduced the total power percentage of the δ wave from 45.5% ± 4.6% to 28.8% ± 9.1% (*t*_(4)_ = 5.289, *P* = 0.0061, *n* = 5 per group), but increased power percentages of the α wave (8.0% ± 1.0% *vs* 10.1% ± 1.1%, *t*_(4)_ = 4.059, *P* = 0.0154), the β wave (11.0% ± 2.3% *vs* 18.2% ± 4.8%, *t*_(4)_ = 4.136, *P* = 0.0144), and the γ wave (12.0% ± 2.8% *vs* 19.5% ± 7.0%, *t*_(4)_ = 3.231, *P* = 0.0319, Fig. 3I).

However, optical inhibition of LHA glutamatergic neurons in the NpHR group did not cause any significant change of burst-suppression oscillation under 1.0% isoflurane anesthesia (Fig. 4D–F). During the mild level of anesthesia induced by 0.8% isoflurane, optical inhibition slightly decreased the power percentage of the β wave from 11.9% ± 1.5% to 11.0 ± 1.3% (t_(4)_ = 3.153, *P* = 0.0344, *n* = 5 per group, Fig. 4G–I).

### Optical activation of LHA glutamatergic terminals in the LHb promotes arousal from isoflurane anesthesia

**Fig. 5:**
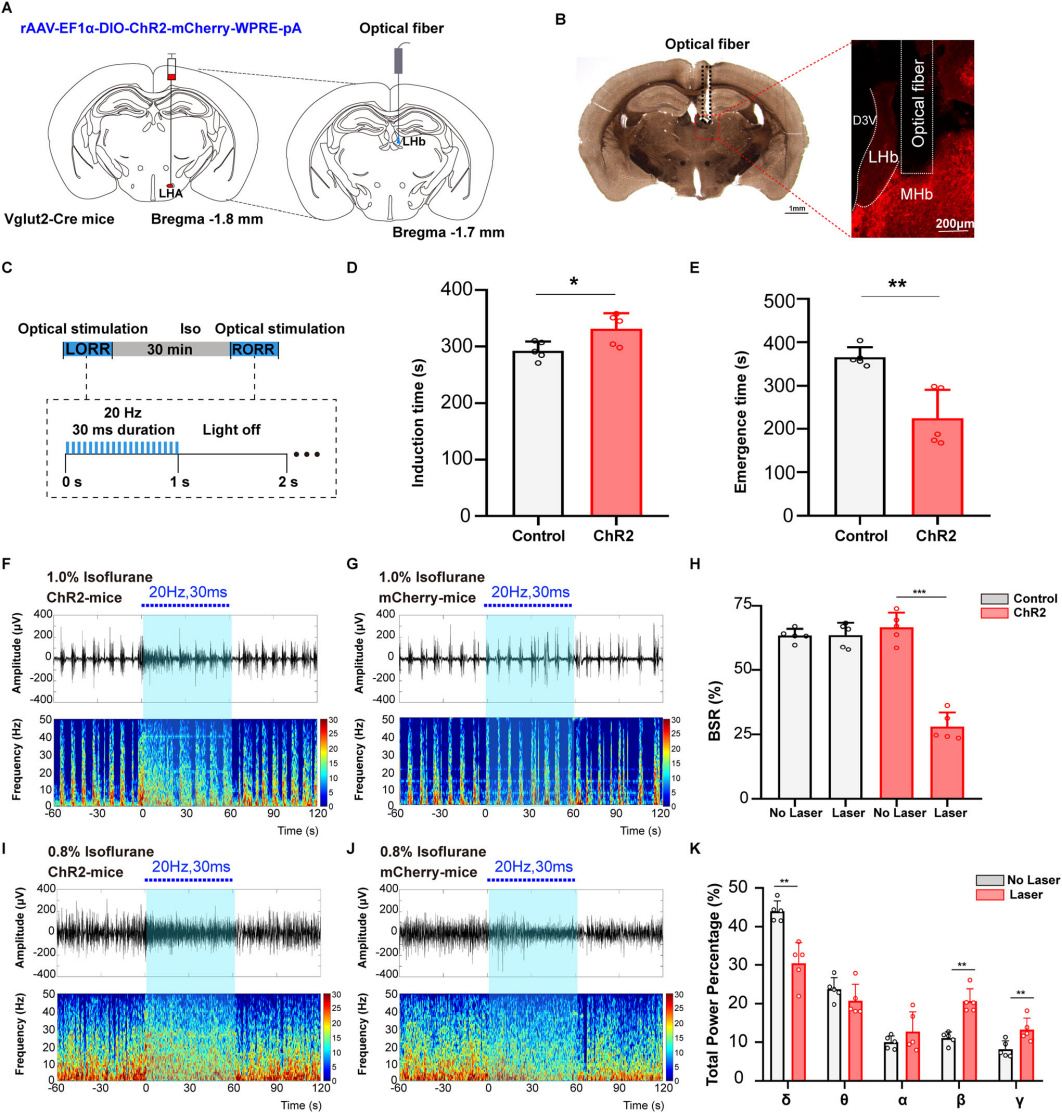
Optical activation of LHA glutamatergic terminals in the LHb regulates the anesthesia behavior and EEG pattern. **A** Diagram showing the injection of excitatory optogenetic virus (ChR2) into the LHA and optical fiber insertion into the LHb of Vglut2-Cre mice. **B** Histological and fluorescent images showing the unilateral location of optical fiber in the LHb. **C** Protocol showing the photostimulation of LHA glutamatergic terminals in the LHb during isoflurane anesthesia. **D**, **E** Effects of activating the glutamatergic LHA–LHb projection on the induction time (**D**) and emergence time (**E**) under isoflurane anesthesia. **F**, **G** Representative EEG traces (above) and corresponding power spectra (below) before, during, and after optical activation under 1.0% isoflurane anesthesia in the ChR2 group (**F**) and the Control (**G**) group. **H** Comparison of BSR before and during optical stimulation of LHA glutamatergic terminal in the LHb between the Control (white) and ChR2 (red) groups. **I**, **J** Changes in spectral power percentage for 1 min before and after optical activation in the ChR2 group (**I**) and the Control (**J**) group under 0.8% isoflurane anesthesia. **K** Statistics of spectral power percentage change in the two groups. Data are shown as the mean ± standard deviation, *n* = 5 per group; *P <0.05, **P <0.01, ***P <0.001; BSR, burst-suppression ratio; D3V, dorsal 3rd ventricle; Iso, isoflurane; LHA, lateral hypothalamic area; LHb, lateral habenula; LORR, loss of righting reflex; MHb, medial habenula; RORR, recovery of righting reflex.

We unilaterally injected the excitatory optogenetic virus (ChR2) into the LHA, and placed an optical fiber into the LHb of Vglut2-Cre mice to specifically activate the LHA glutamatergic terminals in the LHb (Fig. 5A)., and examined the fiber location in each animal after experiments (Fig. 5B). We gave continuous stimulation during the induction and emergence periods of anesthesia (Fig. 5C). Optogenetic activation of LHA glutamatergic terminals in the LHb slightly prolonged the induction time from 292.8 ± 16.1 s to 331.2 ± 28.0 s (*t*_(8)_ = 2.656, *P* = 0.0290, *n* = 5 per group, Fig. 5D), while markedly reducing the emergence time from 366.2 ± 22.2 s to 224.6 ± 66.0 s (*t*_(8)_ = 4.545, *P* = 0.0019, *n* = 5 per group, Fig. 5E).

During the maintenance of 1.0% isoflurane anesthesia, activation of the LHA-LHb glutamatergic projection clearly reduced the burst suppression pattern of EEG recordings (Fig. 5F) compared with the control group (Fig. 5G). The BSR in the ChR2 group significantly decreased from 66.7% ± 5.8% to 28.0% ± 5.6% (*t*_(4)_ = 12.67, *P =* 0.0002, *n* = 5 per group), as shown in Fig. 5H. Furthermore, under lighter anesthesia maintained by 0.8% isoflurane, optical activation of LHA glutamatergic terminals in the LHb changed the power of multiple frequency bands in the ChR2 group (Fig. 5I), but not the control group (Fig. 5J). The power percentage of the δ wave in the ChR2 group declined from 44.0% ± 2.7% to 30.5% ± 5.4% (*t*_(4)_ = 5.764, *P* = 0.0045, *n* = 5 per group), while the power percentages of the β wave (11.1% ± 1.6% *vs* 20.7% ± 3.2%, *t*_(4)_ = 4.816, *P* = 0.0085) and the γ wave (8.3% ± 2.1% *vs* 13.3% ± 3.0%, *t*_(4)_ = 5.418, *P* = 0.0056, Fig. 5K) increased.

### Optical inhibition of LHA glutamatergic projections to the LHb delays the emergence from isoflurane anesthesia

**Fig. 6:**
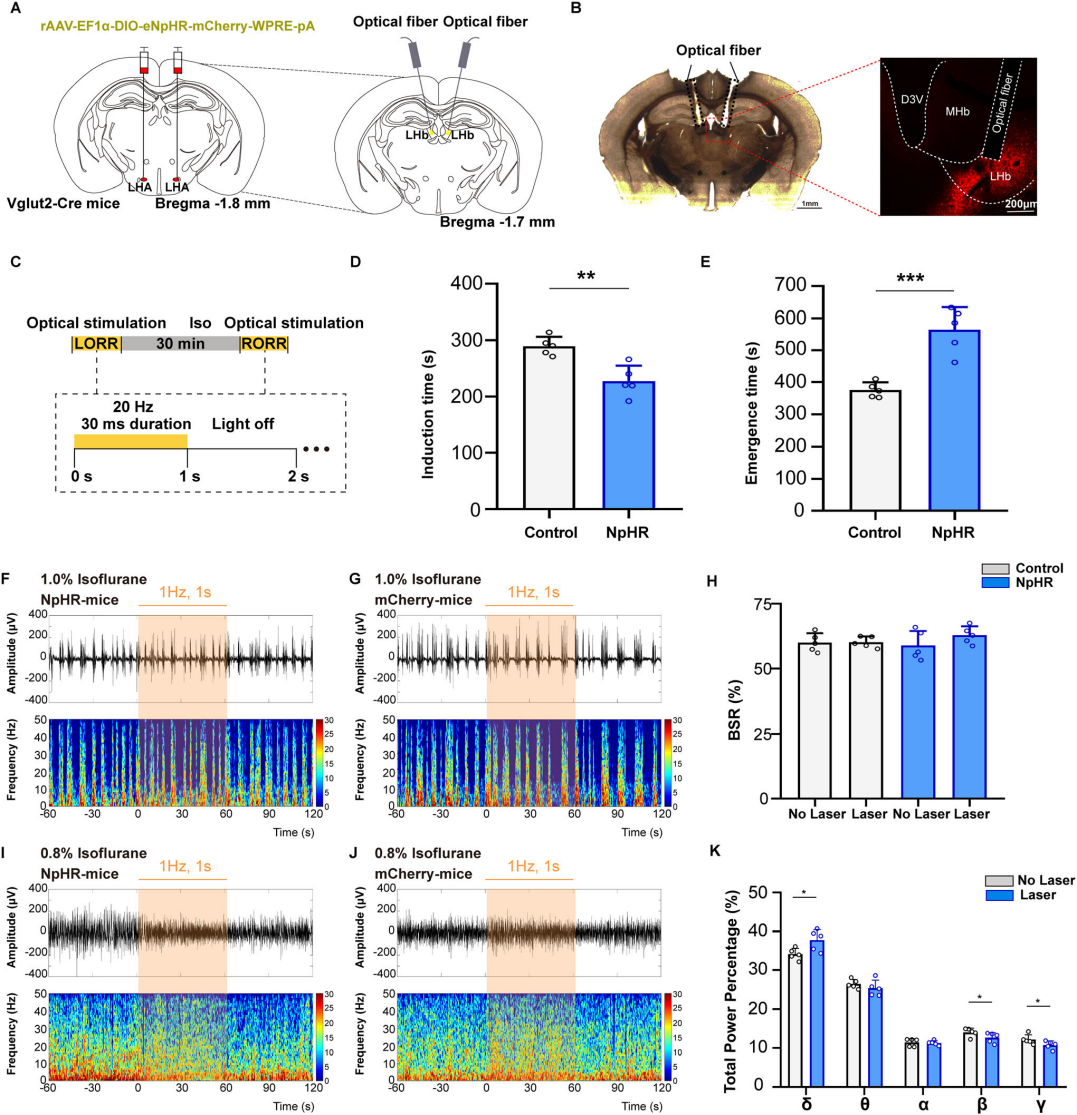
Optical inhibition of LHA glutamatergic projections to LHb delays emergence from isoflurane anesthesia. **A** Diagram showing injection of the inhibitory optogenetic virus (NpHR) into the LHA followed by optical fiber implantation in the LHb of Vglut2-Cre mice. **B** Histological and fluorescent images showing the bilateral location of optical fibers in the LHb. **C** Protocol of photostimulation during the righting reflex test under isoflurane anesthesia. **D**, **E** Effects of inhibiting the glutamatergic LHA–LHb projection on the induction time (**D**) and emergence time (**E**) under isoflurane anesthesia. **F**, **G** Representative EEG traces (above) and corresponding power spectra (below) before, during, and after optical inhibition under 1.0% isoflurane anesthesia in the NpHR group (**F**) and the Control (**G**) group. **H** Comparison of BSR before and during optical stimulation of LHA glutamatergic terminals in the LHb between the Control (white) and NpHR (blue) groups. **I**, **J** Changes in the spectral power percentage for 1 min before and after optical inhibition in the NpHR group (**I**) and the Control (**J**) group under 0.8% isoflurane anesthesia. **K** Statistics of spectral power percentage change in the two groups. Data are shown as the mean ± standard deviation, *n* = 5 per group; *P <0.05, **P <0.01, ***P <0.001; BSR, burst-suppression ratio; D3V, dorsal 3rd ventricle; Iso, isoflurane; LHA, lateral hypothalamic area; LHb, lateral habenula; LORR, loss of righting reflex; MHb, medial habenula; RORR, recovery of righting reflex.

To specifically inhibit the LHA glutamatergic terminals in the LHb, inhibitory optogenetic virus (NpHR) was bilaterally injected into the LHA, followed by the implantation of optic fibers into the LHb of Vglut2-Cre mice (Fig. 6A). The fiber locations are shown in Fig. 6B. Yellow laser light was delivered during the induction and emergence periods of isoflurane anesthesia (Fig. 6C). Optogenetic inhibition of LHA glutamatergic terminals in the LHb shortened the induction time from 289.6 ± 16.6 s to 227.4 ± 27.5 s (*t*_(8)_ = 2.656, *P* = 0.0025, *n* = 5 per group, Fig. 6D), and prolonged the emergence time from 375.2 ± 23.9 s to 564.6 ± 70.2 s (*t*_(8)_ = 5.713, *P* = 0.0004, *n* = 5 per group, Fig. 6E).

Consistent with the local inhibition of LHA glutamatergic cell bodies, inhibition of the LHA glutamatergic projections to the LHb did not affect the BSR during deep (1.0%) isoflurane anesthesia, as shown in Fig. 6F–H. Under the lighter anesthesia induced by 0.8% isoflurane (Fig. 6I–K), inhibition of the LHA glutamatergic projection to the LHb increased the power percentage of the δ wave from 34.1% ± 1.5% to 37.8% ± 2.8% (*t*_(4)_ = 3.035, *P* = 0.0386, *n* = 5 per group), while reducing the power percentage of the β wave (14.0% ± 1.1% *vs* 12.7% ± 1.3%, *t*_(4)_ = 4.393, *P* = 0.0118) and the γ wave (12.1% ± 1.3% *vs* 10.8% ± 1.0%, *t*_(4)_ = 3.089, *P* = 0.0366).

## Discussion

In this study, we found that pharmacogenetic ablation of LHA glutamatergic neurons did not abolish the effect of isoflurane anesthesia, but accelerated the transition from wakefulness to the anesthetic state and delayed the recovery from anesthesia to wakefulness, suggesting a potential effect of LHA glutamatergic neurons in the regulation of both the induction and emergence of anesthesia. Modulation of the activity of LHA glutamatergic neurons using the chemogenetic approach also induced a time shift in the transitions from wakefulness to anesthesia and anesthesia to arousal. Transient optical activation of LHA glutamatergic neurons reduced the depth of anesthesia maintenance in terms of BSR reduction and EEG power redistribution. Furthermore, the optical activation of LHA glutamatergic terminals in the LHb not only decreased the depth of anesthetic maintenance, but also facilitated the transition from anesthesia to arousal, while inhibition of LHA glutamatergic terminals in the LHb had the opposite effect.

As a functionally and anatomically complex region, the LHA contains orexinergic, MCH, GABAergic, and glutamatergic neurons, which regulate many physiological and behavioral processes. The orexinergic and MCH neurons are exclusively restricted to the LHA, playing opposite roles in the regulation of sleep. Orexinergic neurons are wake-active and necessary for maintaining wakefulness [24, 25]. By contrast, the MCH neurons discharge in a reciprocal manner to orexinergic neurons and promote sleep [26, 27]. Activation of the GABAergic neurons in the LHA has been shown to produce sustained wakefulness [7]. With regard to anesthesia, activation of orexinergic neurons facilitates emergence from isoflurane anesthesia [28]. Blocking the orexin-1 receptor increases the emergence time, without influencing the induction time [29]. Our previous research confirmed that this arousal facilitation of the orexin system is partially mediated by their projections to the basal forebrain and ventral tegmental area [3–5, 23]. A large number of orexinergic neurons (>50%) express vesicular glutamate transporters [30], suggesting they co-release glutamate from their terminals [31, 32]. It is reasonable to expect that LHA glutamatergic neurons share a similar regulatory effect on anesthesia with orexinergic neurons. Surprisingly, LHA glutamatergic neurons participated in the whole process of isoflurane anesthesia, not only the emergence period, but also the induction period and anesthesia maintenance. In particular, excitation of LHA glutamatergic neurons shortened the emergence time, and prolonged the induction time of isoflurane anesthesia. It is worth noting that the shortening of emergence time was much greater than the delay of induction (122.2 ± 50.9 s decrease of time to RORR *vs* 40.5 ± 26.2 s increase of time to LORR, *t*_(10)_ = 3.496, *P =* 0.0058, Fig. 2H, G). This implies the potential participation of non-orexin glutamatergic neurons in the regulation of isoflurane induction. In fact, single-cell transcriptomic tests have shown that LHA glutamatergic neurons can be clustered into 15 distinct populations [33]. This heterogeneity probably determines their diverse functions in the regulation of isoflurane anesthesia, while also suggesting they have multiple downstream nuclei.

The nucleus of the LHb, receiving dense projections from the LHA glutamatergic neurons [34], has been reported to participate in propofol anesthesia [17] and food consumption [16]. In the current study, optical activation of LHA glutamatergic terminals in the LHb induced a reduction of emergence time and a shift of the EEG pattern to arousal. However, the pro-arousal effect of LHA glutamatergic terminals in the LHb was not as strong as in their cell bodies. Specifically, the BSR decrease by optical activation of LHA glutamatergic projections to the LHb was less than that by the photostimulation of LHA glutamatergic cell bodies (38.7% ± 6.8 % *vs* 52.9% ± 3.2%, *t*_(8)_ = 4.210, *P =* 0.0030, Figs 3F, 5H). Besides the LHb, anterograde mapping has helped to identify the other down-stream nuclei of LHA glutamatergic neurons, such as the lateral septum and the anterodorsal thalamus [15]. Previous studies have also demonstrated that the prefrontal cortex (PFC) is an important target of the LHA [35], and a recent study revealed that cholinergic stimulation of the PFC is sufficient to restore the level of consciousness and wake-like behavior under constant sevoflurane anesthesia [36]. Therefore, the activation of multiple downstream nuclei could be an important reason for the stronger pro-emergence effect of LHA glutamatergic cell bodies. On the other hand, LHA glutamatergic neurons also innervate orexinergic neurons directly or indirectly. Previous studies have found that LHA orexinergic neurons express glutamate receptors [37]. Application of glutamate receptor agonists (AMPA and NMDA) induces the depolarization of orexinergic neurons, while their antagonists (CNQX and AP-5) have the opposite effect [38]. Furthermore, microinjection of l-glutamic acid into the LHA contributes to the promotion of arousal and the suppression of REM and NREM sleep [14]. Similarly, local perfusion of NMDA into the LHA dose-dependently increases the time of active waking, but this effect is significantly attenuated in orexinergic neuron knockout mice [39]. All this evidence suggests that the pro-arousal effect of LHA glutamatergic neurons is partially achieved by their projection to the LHb, but may also be mediated through the long projections to other nuclei and local micro-projections to orexinergic neurons.

Of note, optical inhibition of LHA glutamatergic neurons or their terminals in the LHb did not change the burst suppression pattern of EEG under deep anesthesia (1% isoflurane), and only slightly changed the power spectrogram under light anesthesia (0.8% isoflurane). We speculate that the activity of LHA glutamatergic neurons is severely inhibited during the maintenance of deep anesthesia, so further inhibition of these neurons could hardly produce a change in the EEG pattern. With the help of glutamate sensors, it has been reported that glutamate in the hypothalamus increases in active waking, but decreases in NREM sleep [13]. Whether the LHA-released glutamate is reduced during anesthesia transition remains to be explored.

This study contains some limitations. Although LHb neurons are predominantly glutamatergic [40], a small subset express glutamic acid decarboxylase 2 [41–43], which is recognized as a GABA marker. Neurons in the LHb respond differently to the activation of LHA glutamatergic neurons [16]. Therefore, the specific neuronal types in the LHb innervated by the LHA glutamatergic projections remain to be investigated.

In conclusion, our study identified the involvement of LHA glutamatergic neurons in the regulation of anesthesia. This regulatory effect of LHA glutamatergic neurons may be partially mediated by their projections to the LHb during anesthesia.
